# Thoracoscopic partial lung resection following pneumonectomy: a report of three cases

**DOI:** 10.1186/s13019-019-1008-6

**Published:** 2019-11-04

**Authors:** Hidenori Goto, Mingyon Mun, Shohei Mori, Joji Samejima, Yosuke Matsuura, Masayuki Nakao, Hirohumi Uehara, Ken Nakagawa, Sakae Okumura

**Affiliations:** Department of Thoracic Surgical Oncology, Japanese Foundation for Cancer Research, Cancer Institute Hospital, 3-8-31, Ariake, Koto, Tokyo, Tokyo 135-8550 Japan

**Keywords:** Thoracoscopic partial lung resection, Pneumonectomy, Tumor, Thoracoscopic surgery

## Abstract

**Background:**

The prognosis of patients who undergo unilateral pneumonectomy and subsequently develop a contralateral pulmonary tumor can be improved by tumor resection. Thus, surgery is a treatment option if the patient’s pulmonary function and performance status are satisfactory. To date, there have been only few cases reporting thoracoscopic lung resection for pulmonary tumor after contralateral pneumonectomy because of the difficulty in respiratory management during surgery. Thoracoscopic surgery requires the maintenance of the operative field to allow the lung to collapse, and in partial lung resection we need to identify tumor localization. The identification of a tumor lesion just inferior to the pleura is easy; however, the identification of a tumor lesion in the deep parts is difficult. The tumor in the deep part of the lung segments can be easily located if the tumor-affected lobe is allowed to completely collapse. Therefore, ventilation technique should be modified according to the tumor localization.

**Case presentation:**

Here, we report three cases of thoracoscopic partial lung resections for pulmonary tumors that developed after contralateral pneumonectomy. Intermittent manual ventilation using a tracheal tube was performed in two cases with a lesion just inferior of the pleura. The tumors in both patients were resected using automatic suturing devices while arresting manual ventilation. The affected lobe was allowed to collapse using a bronchial blocker in one of the cases with a lesion in the deep part. Furthermore, she had contralateral pneumothorax with bullae on the right upper and lower lobes of the lung. The tumor in the deep part of the lung segment and ruptured bullae were easily located and resected using automatic suturing devices. The hemodynamic status of the patients was stable, and the intra- and postoperative courses were uneventful.

**Conclusions:**

Our cases demonstrate that thoracoscopic lung resection after contralateral pneumonectomy can be performed if intermittent manual ventilation is utilized when the tumor is located just inferior to the pleura and if selective double ventilation using an intrabronchial blocker is utilized when the tumor is located in the deep part.

## Background

The prognosis of patients who undergo unilateral pneumonectomy and subsequently develop a contralateral pulmonary tumor can be improved by tumor resection. Thus, surgery is a treatment option if the patient’s pulmonary function and performance status are satisfactory. However, respiratory management during surgery is difficult because of the limited lung volume. Thoracoscopic surgery is a less invasive procedure than thoracotomy, and partial resection is indicated for tumors if a sufficient resection margin can be obtained. Thoracoscopic surgery requires the maintenance of the operative field to allow the lungs to collapse during the procedure of maintaining cardiopulmonary function under intraoperative anesthesia. The identification of a tumor lesion just inferior to the pleura is easy; however, the identification of a tumor lesion in the deep parts is difficult. Therefore, ventilation technique needs to allow the lungs to completely collapse for the identification of tumor localization. Ventilation technique will be modified according to the tumor localization. Here, we present useful techniques for thoracoscopic partial lung resections.

## Case presentation

**Fig. 1:**
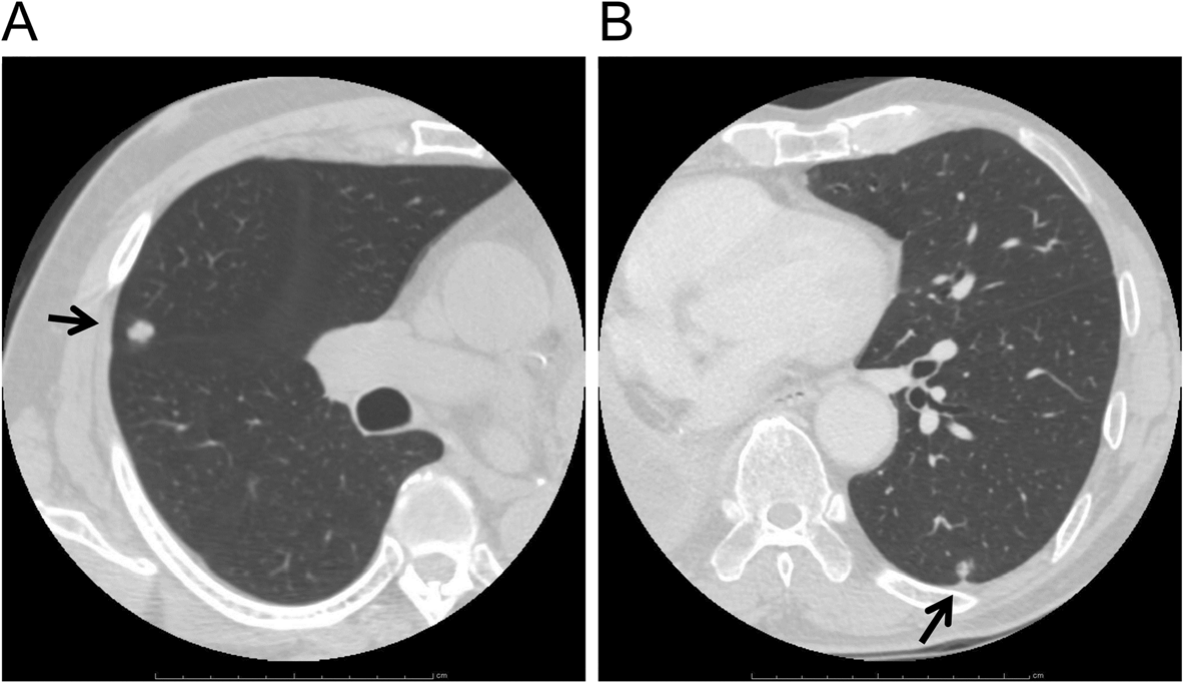
Preoperative computed tomography images The arrows indicate the tumor location. In one case, the tumor just inferior of the pleura at the right S4 lung segment is visualized in the horizontal plane (**a**). In the second case, the tumor just inferior of the pleura at the left S10 lung segment is visualized in the horizontal plane (**b**).

**Fig. 2:**
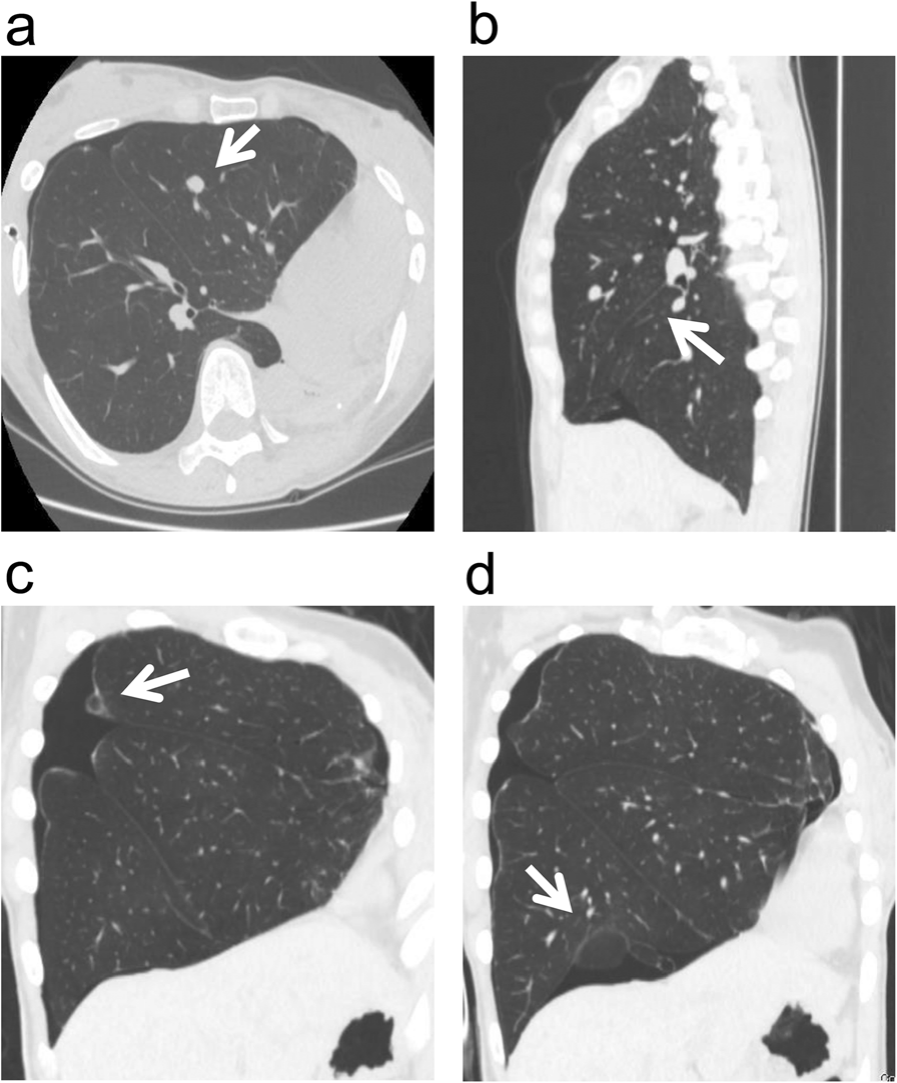
Computed tomography (CT) image of the chest. CT image (**a**, axial view; **b**, sagittal view; **c** and **d**, coronal views) reveals a tumor in the deep part of the right S4 lung segment (**a** and **b**) and bullae on the right upper and lower lobes of the lung (**c** and **d**).

**Fig. 3:**
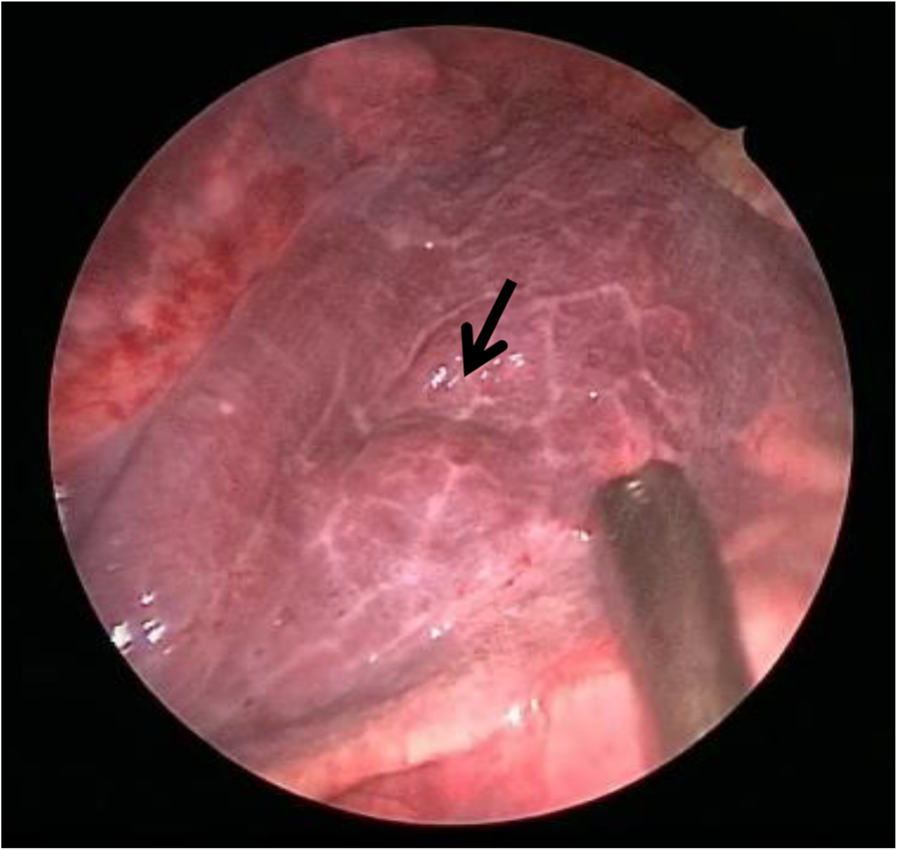
Thoracoscopic view of intraoperative screen images with a bronchial blocker to collapse the affected lobe demonstrates a satisfactory collapse of the lobe because the bulging region of the tumor in the deep part of the lung segment is exposed (black arrow)

We report three cases of thoracoscopic partial lung resections for pulmonary tumors that developed after a contralateral pneumonectomy. In one case, a 69-year-old man who had undergone a left pneumonectomy, 4 months prior for multiple metastatic pulmonary tumors originating from colon cancer, underwent a thoracoscopic partial lung resection for a tumor just beneath the pleura in his right S4 lung segment (Fig. 1a). In the second case, a 66-year-old man who had undergone a right pneumonectomy 1 year prior for lung cancer underwent a thoracoscopic partial lung resection for a tumor just beneath the pleura in his left S10 lung segment (Fig. 1b). The patients were diagnosed with oligometastasis because they were found to have a retrospectively increased single nodule and no other lesions in the preoperative study, including [^18^F]-fluorodeoxyglucose positron emission tomography. Preoperative pulmonary function testing revealed a forced expiratory volume in 1 s (FEV1) of 1.35 L (60% predicted) and 1.79 L (72% predicted), a forced vital capacity (FVC) of 1.52 L (47% predicted) and 2.38 L (72% predicted), a FEV1/FVC ratio of 89 and 75% predicted, respectively. In these two cases, intermittent manual ventilation via a tracheal tube using high-concentration O_2_ (66 and 75%, respectively) was required, and the adequacy of the CO_2_ exchange was confirmed through the measurement of end-tidal CO_2_. The tumors in both patients were readily located just beneath the pleura and resected with Endo-GIA 60 (Tyco Healthcare Japan, Tokyo, Japan) while arresting manual ventilation. Total surgery time was 44 and 21 min, respectively. In the third case, a 43-year-old woman who had undergone a left wedge pneumonectomy 8 years prior for a tracheobronchial adenoid cystic carcinoma underwent a thoracoscopic partial lung resection for contralateral pneumothorax and a tumor in the deep part of her right S4 lung segment (Fig. 2a and b). Bullae on the right upper and lower lobes of the lung (Fig. 2c and d) and a deeply located tumor required excision. While breathing room air, the pulse oximeter O_2_ saturation of the patients was 97% with continuous thoracic drainage (− 10 cm H_2_O), and preoperative arterial blood gas analysis showed pH of 7.41, PaCO_2_ of 43.0 mmHg, and PaO_2_ of 78.1 mmHg. The patient was diagnosed with oligometastasis because she was found to have a retrospectively increased single nodule and no other lesions on computed tomography scans. The tumor was excised, and bullectomy was performed immediately. Preoperative selective double ventilation of the right upper and lower lobes using a Coopdech Bronchial Blocker BBT-A3060 (Daiken Medical, Osaka, Japan) was performed to facilitate these resections, collapse the tumor-affected lobe, and facilitate the resection of the tumor. Ventilation using high-concentration O_2_ (84%) was required, and the adequacy of the CO_2_ exchange was confirmed by the measurement of end-tidal CO_2_. Despite artificial ventilation of the other lobes in the same period, the operative field was maintained to allow the lung to collapse. A ruptured bulla at the right S2 lung segment and the other bullae at the right S8 lung segment were found and resected with Endo-GIA 60. Because the lungs were allowed to completely collapse, the tumor in the deep part of lung segment was readily located (Fig. 3) and resected with Endo-GIA 60. Total operation time was 75 min. In all cases, a sufficient resection margin (>tumor greatest dimension) was obtained with negligible blood loss. The hemodynamic statuses were stable and the intra- and postoperative courses were uneventful; all patients were reported to be ambulatory upon discharge from the hospital approximately 7 days after surgery. To date, all the patients are alive. The third patient is suspected of the presence of brain metastasis 35 months after lung surgery. It has been 10 years and 36 months, respectively, since the surgery of the other two patients, and neither have had a tumor recurrence.

## Discussion and conclusions

The ACCP guidelines of 2013 recommend that in patients with contralateral lobe nodule, evaluation of extrathoracic metastases and invasive evaluation to rule out mediastinal node involvement should be performed; furthermore, they recommend resection of each lesion. Fernandez et al. [1] stated that curative intent for oligometastatic disease to organs other than the lungs, brain, and adrenal glands should be considered on a case-to-case basis. Furthermore, most data to support locally directed treatment, such as stereotactic ablative radiotherapy, for oligometastases are from retrospective institutional reports [2]. Consequently, pulmonary resection of oligometastasis should be considered in patients with adequate pulmonary function. When patients undergo unilateral pneumonectomy and subsequently develop a contralateral pulmonary tumor, we should take cognizance of the patient’s pulmonary function, whether or not the tumor is metachronal lung cancer, and whether or not it is a metastatic lesion when determining the operative method. Toufektzian et al. [3] stated that pulmonary resection for metastatic or metachronous diseases can be performed with acceptable morbidity and low mortality in appropriately selected patients who have previously undergone pneumonectomy. Donington et al. [4] stated that limited resections should be the method of choice after a pneumonectomy. Wedge resections with negative margins are the preferred procedure for peripheral pulmonary tumors. Conversely, central pulmonary tumors would benefit more from segmentectomy [5]. Thoracoscopic surgery is increasingly used with the purported benefits of less postoperative pain and respiratory dysfunction, leading to faster recovery and hospital discharge [6], but requires the maintenance of the operative field so that the lungs are allowed to collapse during the procedure to maintain cardiopulmonary function under intraoperative anesthesia. Although few in number, previous reports after a contralateral pneumonectomy have included thoracoscopic surgery with high-frequency jet ventilation as a treatment option for pulmonary tumors [7] and with selective double ventilation using an intrabronchial blocker as a treatment option for the bulla in close proximity to the lung tumor [8]. However, there have been few cases reporting thoracoscopic partial lung resection according to the tumor localization. It is easy to identify tumor lesion just inferior of the pleura; however, identification is difficult for tumor lesion in the deep part. Therefore, ventilation technique needs to allow the lungs to completely collapse for the identification of tumor localization. Here we report three cases of thoracoscopic partial lung resection for a pulmonary tumor after a contralateral pneumonectomy, and extracorporeal cardiopulmonary support was not needed in any of the cases. When the tumor was located just inferior of the pleura, the resection was performed by stopping manual ventilation with high-concentration O_2_. When the tumor in the deep part was difficult to locate due to the hyperinflation of the lung, double-lobe ventilation was performed using a bronchial blocker to collapse the affected lobe. If a satisfactory collapse of the lobe was obtained, the bulge region of the tumor in the deep part of lung segment was exposed, allowing easy and accurate tumor localization.

Our cases demonstrate that the intermittent manual ventilation and selective double ventilation using an intrabronchial blocker enable the use thoracoscopic lung resection for patients after a contralateral pneumonectomy.

## Data Availability

Not applicable.

## Abbreviations

FEV1: Forced expiratory volume in 1 s
FVC: Forced vital capacity
